# Caractere generique du Caoutchouc

**Published:** 1785

**Authors:** 


IV. *Caractere generique du Caoutchouc. Par* M. Richard, *Botaniſte du Roi à Cayenne.* Vide *the* Journal de Phyſique *for Auguſt*, 1785.

THE tree which yields the Caoutchouc, or elaſtic gum, has long been an object of curioſity to botaniſts. It was firſt mentioned by M. de la Condamine*, who had ſeen it growing on the banks of the river of Amazons; and has ſince been deſcribed by M. Freſnau†, a French engineer, who found it to be

* Relation d'un Voyage, &c. 8vo. Paris, 1745.

† Mem. de l'Acad. des Sciences. 4to. Paris, 1751.

be a native of Cayenne; and more lately by M. Aublet *, who gives it the name of *Hevea Guianensis*. Neither of these gentlemen, however, had seen the flowers of the tree, so that its generical character remained undetermined.

The younger Linnæus, from the structure of its fruit, supposed it to be a species of Jatropha †; but M. Richard, who has lately had an opportunity of seeing it in flower, finds that it forms a distinct genus, of which he has communicated the following description to the Editor of the Journal de Physique, in a letter, dated at Cayenne, January 7th, 1785:

" Caoutchouc.

" Caracter Genericus.

" Flores. *Mares* numerosi et unicus *femina* " terminalis in eodem receptaculo.

" Marium.

" Calyx. Globoso-campanulatus, semiquin- " que fidus; dentibus erectis, acutis, " marginibus introflexis.

" Stamina. In fundo calycis surgit colum- " nula ipso tertia parte brevior, cylin-

* Hist. des Plantes de la Guiane Francoise. 4to. Paris, 1774.

† Supplement. Plant. p. 422.

" dracea,

" dracea, gerens *antheras* quinque, in-

" fra ipsius apicem immediate et longi-

" tudinaliter dorso suo adnexas. Hæ

" sunt subovatæ, apice subemarginatæ,

" basi acuminulatæ, biloculares; locu-

" lis bivalvibus: *pollinis* particulæ

" ovatæ.

" Feminarum.

" Calyx. Subpyriformi-campanulatus; den-

" tibus quinque acutis, recurvo-paten-

" tibus. Circumscisse à basi discedit

" et cadit.

" Pistillum. Calyce duplo brevius: *Ger-*

" *men* subconoideo-globosum: *Stigmata*

" tria, apici istius immediate adnata,

" crassiuscula, depresso-biloba.

" Fructus. *Capsula* magna, tricocca: *Peri-*

" *carpium* tenue, fibrosum; adhæse ves-

" tiens *Nucem* magnam tricoccam, du-

" rissimam, osseam, crassam, apice de-

" pressam, basi excavatam et perfora-

" tam, tribus rimis inter loculamenta

" pertusam. Discedit in tres loculos

" subovatos, elastice bivalvos; valvis

" sub auriculæ formibus. In singulo

" *semen* unicum (aut duo, Aublet.) sub-

" ovatum,

"ovatum, hinc lineola depreſſa lon-

"gitudinali leniter exaratum, griſeo

"flaveſcens, fuſco-maculoſum."

